# Association of anti-glomerular basement membrane antibody disease with dermatomyositis and psoriasis: case report

**DOI:** 10.1590/S1516-31802010000500012

**Published:** 2010-09-02

**Authors:** Natália Pereira Machado, Cintia Zumstein Camargo, Ana Cecília Diniz Oliveira, Ana Letícia Pirozzi Buosi, Mário Luiz Cardoso Pucinelli, Alexandre Wagner Silva de Souza

**Affiliations:** I MD. Medical resident, Rheumatology Division, Universidade Federal de São Paulo (Unifesp), São Paulo, Brazil.; II MD, PhD. Medical resident, Rheumatology Division, Universidade Federal de São Paulo (Unifesp), São Paulo, Brazil.

**Keywords:** Psoriasis, Dermatomyositis, Anti-glomerular basement membrane disease, Antibodies, antineutrophil cytoplasmic, Glomerulonephritis, Psoríase, Dermatomiosite, Doença antimembrana basal glomerular, Anticorpos anticitoplasma de neutrófilos, Glomerulonefrite

## Abstract

**CONTEXT:**

Anti-glomerular basement membrane (anti-GBM) antibody syndrome is characterized by deposition of anti-GBM antibodies on affected tissues, associated with glomerulonephritis and/or pulmonary involvement. This syndrome has been described in association with other autoimmune disorders, but as far as we know, it has not been described in association with dermatomyositis and psoriasis.

**CASE REPORT:**

A 51-year-old man with a history of dermatomyositis and vulgar psoriasis presented with a condition of sensitive-motor polyneuropathy of the hands and feet, weight loss of 4 kg, malaise and fever. On admission, he had been making chronic use of cyclosporin and antihypertensive drugs for three months because of mild arterial hypertension. Laboratory tests showed anemia and leukocytosis, elevated serum urea and creatinine and urine presenting proteinuria, hematuria, leukocyturia and granular casts. The 24-hour proteinuria was 2.3 g. Renal biopsy showed crescentic necrotizing glomerulonephritis with linear immunoglobulin G (IgG) deposits on the glomerular basement membrane by means of direct immunofluorescence, which were suggestive of anti-GBM antibodies. The patient was then treated initially with methylprednisolone and with monthly cyclophosphamide in the form of pulse therapy.

## RESUMO

**CONTEXTO:**

A síndrome do anticorpo anti-membrana basal glomerular (anti-MBG) é caracterizada pela deposição de anticorpos anti-MBG em tecidos afetados, associada à glomerulonefrite e/ou ao envolvimento pulmonar. Essa síndrome já foi descrita em associação a outras doenças autoimunes, mas até onde conhecemos, não há relatos de sua associação com dermatomiosite e psoríase.

**RELATO DE CASO:**

Um homem de 51 anos com antecedentes de dermatomiosite e psoríase vulgar apresentou quadro de polineuropatia sensitivo-motora de mãos e pés, perda de 4 kg, adinamia e febre. À admissão estava em uso crônico de ciclosporina e de anti-hipertensivos há três meses devido a hipertensão arterial leve. Exames laboratoriais mostraram anemia e leucocitose, creatinina e ureia séricas elevadas e urina com proteinúria, hematúria, leucocitúria e cilindros granulosos. A proteinúria de 24 horas foi de 2,3 g. A biópsia renal revelou uma glomerulonefrite crescêntica necrotizante com depósitos lineares de imunoglobulina G (IgG) na MBG à imunofluorescência, sugestivos de anticorpos anti-MBG. O paciente foi então tratado inicialmente com metilprednisolona e com ciclofosfamida mensalmente na forma de pulsoterapia.

## INTRODUCTION

Dermatomyositis is an autoimmune disease that is characterized by involvement of proximal musculature and skin. There are a few cases involving the kidneys, such as membranous and mesangial proliferative glomerulonephritis.^1^ Anti-glomerular basement membrane (anti-GBM) antibody disease or Goodpasture’s disease is caused by linear deposition of anti-GBM antibodies on the glomerular and/or alveolar basement membrane. It is called Goodpasture’s syndrome when it leads to glomerulonephritis and lung hemorrhage. This is a rare condition, with annual incidence of about one case per million people, and is more prevalent among white males and, most commonly, in the third and seventh decades of life. There is an association with human leukocyte antigen (HLA) DR15 or DR4.^2-4^

In this study, we present one patient with dermatomyositis and vulgar psoriasis who developed anti-GBM disease and was positive for perinuclear antineutrophil cytoplasmic antibodies (p-ANCA).

## CASE REPORT

In October 2008, a 51-year-old man presented at our hospital complaining of a six-day history of weakness of left foot dorsiflexion. He had been feeling burning pain, paresthesia and edema of the distal legs and hands bilaterally for the previous 40 days. He also reported polyarthralgia and weight loss of 4 kg over this period.

He had a past history of dermatomyositis fulfilling the Bohan and Peter criteria (proximal muscle weakness, typical rash, elevated serum muscle enzymes and characteristic muscle biopsy abnormalities)^5^ and biopsy-proven vulgar psoriasis, diagnosed ten and six years earlier, respectively. At that time, he was positive for antinuclear antibodies (ANA), with a fine speckled pattern at a 1/320 titer and negative for anti-extractable nuclear antigens (anti-ENA). He also reported presenting corticosteroid-induced diabetes over a three-month period nine years earlier, systemic hypertension for the last six months and previous tobacco use. He denied alcohol and drug abuse. The patient was taking cyclosporin and low doses of losartan, amlodipine and hydrochlorothiazide. On admission, his body temperature was 37 °C and blood pressure was 145/90 mmHg. Physical examination revealed lower-limb edema and cutaneous erythematous-desquamative lesions on his wrists, elbows and lower back area. He also had severe left-foot weakness due to dorsiflexion, and hypoesthesia of his hands and lateral left distal leg.

The laboratory tests on admission showed mild anemia (hemoglobin: 11.2 g/dl), leukocytosis (18,000 cells/mm^3^), elevated serum urea (94 mg/dl) and creatinine (2.09 mg/dl) and an abnormal urinalysis with proteinuria, blood, leukocyturia and granular casts. The 24-hour proteinuria was 2.31 g. He also presented elevated erythrocyte sedimentation rate (86 mm/hour) and C-reactive protein concentration (138.15 mg/l). Serological tests for hepatitis B and C and human immunodeficiency virus (HIV) were all negative.

**Figure 1.**
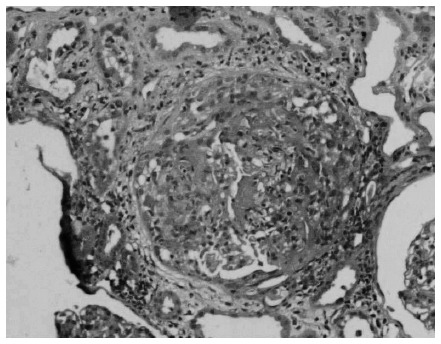
Kidney section stained with hematoxylin and eosin at 20 X magnification, showing crescentic glomerulonephritis with glomerular sclerosis.

**Figure 2.**
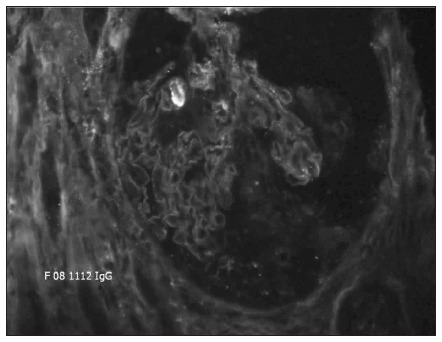
Direct immunofluorescence of kidney biopsy showing linear glomerular deposits (20 X magnification).

Tests for ANA, anti-ENA, anti-Jo1 and anti-PMScl antibodies were all negative, with normal complement, and p-ANCA tested positive using indirect immunofluorescence, with a 1:80 titer. Percutaneous needle renal biopsy showed crescentic necrotizing glomerulonephritis (Figure 1) with linear immunoglobulin G (IgG) deposits on the glomerular basement membrane by means of direct immunofluorescence, which were suggestive of anti-GBM antibodies (Figure 2). Electroneuromyography showed motor-sensitive axonal polyneuropathy in the lower extremities, associated with mononeuropathy of the left fibular nerve. Investigation of paraneoplastic syndrome was negative, including abdominal, chest and prostatic tumors.

A diagnosis of anti-GBM disease was made and treatment was begun with steroid pulse therapy (methylprednisolone 1 g/day for three days) and monthly intermittent intravenous cyclophosphamide (dose: 0.5 g/m^2^). After six months of intravenous cyclophosphamide, the patient persisted with abnormalities of urinary sediment and 24-hour proteinuria of 6 gm. Thus, pulse therapy was replaced by oral cyclophosphamide at a dose of 150 mg/day, which led to partial remission of the renal disease and corticosteroid taper-off within three months.

## DISCUSSION

The patient reported here developed signs of glomerulonephritis within a few years after dermatomyositis had been diagnosed. This led to the clinical suspicion of either renal involvement in this inflammatory myopathy or a syndrome overlapping with another connective tissue disease or systemic vasculitis. Therefore, renal biopsy was indicated, and this confirmed the presence of anti-GBM disease.

The presence of peripheral polyneuropathy was another confounding factor in this case, since this is an unusual manifestation of anti-GBM disease. An association between glomerulonephritis and peripheral polyneuropathy may be encountered in diseases such as ANCA-associated vasculitis, cryoglobulinemia and systemic lupus erythematosus. Moreover, it is well known that Goodpasture’s syndrome classically presents with glomerulonephritis and pulmonary hemorrhage. This may be accompanied by systemic symptoms and, less often, by peripheral neuropathy, arthritis, leukocytoclastic vasculitis and uveitis. In the absence of pulmonary involvement, the diagnosis is referred to as anti-GBM disease.^2-4^

ANCA is found in 30 to 47% of patients with anti-GBM disease and thus, histological evidence is of paramount importance for correct diagnosis of anti-GBM disease, because patients might be erroneously treated for ANCA-associated vasculitis.^3^ The pathogenic effects of anti-GBM was demonstrated by Lerner et al. through the induction of glomerulonephritis in monkeys.^6^ ANCA is also known to have pathogenic effects when binding to myeloperoxidase (MPO) or proteinase 3 (PR3) on the surface of primed neutrophils. It leads to activation of these cells and their migration to and adhesion onto the endothelium, along with release of proteolytic enzymes, proinflammatory cytokines and reactive oxygen species, which eventually causes endothelial cell damage.^7^ Nevertheless, the pathophysiological roles of the two antibodies occurring in patients with anti-GBM disease are not completely understood. Some authors have suggested that ANCA could initiate damage to the glomerular basement membrane, thereby exposing antigens that were previously hidden and triggering the production of anti-GBM antibodies.^8,9^

The management of Goodpasture’s syndrome and anti-GBM disease includes plasmapheresis, in order to remove pathogenic antibodies associated with immunosuppression and thereby inhibit antibody production and avoid rebound hypersynthesis of antibodies following withdrawal of plasmapheresis. High-dose corticosteroids and cyclophosphamide are mostly used, and the duration of therapy with the latter is approximately six months.^2-4^ Notwithstanding these recommendations, our patient was managed successfully with immunosuppressive drugs alone, because of the unavailability of plasmapheresis at the time when he was acutely ill.

Other factors that may have contributed towards a favorable response in this case were the absence of the so-called poor prognostic signs of Goodpasture’s syndrome (e.g. oliguria, advanced kidney fibrosis, serum creatinine > 5.7 mg/dl or dialysis^4^) in our patient. However, patients with both antibodies have similar renal survival to those with anti-GBM antibodies alone.^10^

## CONCLUSIONS

This is the first case in which an association of anti-GBM antibody syndrome with dermatomyositis and psoriasis has been described. We performed an electronic search in the Pubmed and Lilacs (Literatura Latino-Americana e do Caribe em Ciências da Saúde) databases to confirm the lack of previous reports on such an association between dermatomyositis, psoriasis, ANCA and anti-GBM disease in the medical literature (Table 1). This case is useful for reminding clinicians and rheumatologists about the importance of correct investigation and precise diagnosis for patients with autoimmune rheumatic diseases presenting with atypical manifestations. Adequate patient history-taking, physical examination, laboratory tests and auto-antibody investigation, along with histopathological evidence of tissue involvement, should guide the diagnosis.

**Table 1.** Search strategy with MeSH (Medical Subject Headings) terms used by the authors on August 28, 2009

Database	Search strategy	Results
PubMed	Dermatomyositis AND Psoriasis AND (Antibodies, antineutrophil cytoplasmic) AND (Anti-glomerular basement membrane disease)	0
Literatura Latino-Americana e do Caribe em Ciências da Saúde (Lilacs)	Dermatomiosite AND Psoríase AND ANCA AND Doença do anticorpo antimembrana basal glomerular	0
